# Biomechanical characterization of the passive response of the thoracic aorta in chronic hypoxic newborn lambs using an evolutionary strategy

**DOI:** 10.1038/s41598-021-93267-9

**Published:** 2021-07-06

**Authors:** Eugenio Rivera, Claudio Canales, Matías Pacheco, Claudio García-Herrera, Demetrio Macías, Diego J. Celentano, Emilio A. Herrera

**Affiliations:** 1 Departamento de Ingeniería Mecánica, Universidad de Santiago de Chile (USACH), Av. Bernardo O’Higgins 3363, Santiago de Chile, Chile; 2 ICD, P2MN, L2n, Université de Technologie de Troyes, ERL 7004, CNRS, Troyes, France; 3 Departamento de Ingeniería Mecánica y Metalúrgica, Pontificia Universidad Católica de Chile, Av. Vicuña Mackenna 4860, Santiago de Chile, Chile; 4 Laboratorio de Función y Reactividad Vascular, Programa de Fisiopatología, ICBM, Universidad de Chile, Av. Salvador 486, Santiago de Chile, Chile

**Keywords:** Biophysics, Computational biology and bioinformatics, Engineering, Mathematics and computing

## Abstract

The present study involves experiments and modelling aimed at characterizing the passive structural mechanical behavior of the chronic hypoxic lamb thoracic aorta, whose gestation, birth and postnatal period were carried at high altitude (3600 masl). To this end, the mechanical response was studied via tensile and pressurization tests. The tensile and pressurization tests measurements were used simultaneously to calibrate the material parameters of the Gasser–Holzapfel–Ogden (GHO) hyperelasctic anisotropic constitutive model through an analytical-numerical optimization procedure solved with an evolutionary strategy that guarantees a stable response of the model. The model and procedure of calibration adequately adjust to the material behavior in a wide deformation range with an appropriate physical description. The results of this study predict the mechanical response of the lamb thoracic aorta under generalized loading states like those that can occur in physiological conditions and/or in systemic arterial hypertension. Finally, the novel use of the evolutionary strategy, together with the set of experiments and tools used in this study, provide a robust alternative to validate biomechanical characterizations.

## Introduction

Pregnancy and birth under hypobaric hypoxia as seen in high-altitude populations increase fetal and neonatal complications^1,2^. The most common cardiovascular complications are pulmonary arterial hypertension of the newborns (PAHN) and right ventricular remodelling. These conditions are associated with physiological changes and structural alterations of the blood vessels such as an increased reactivity and remodeling^3–6^, involving geometric changes in the structure of the arterial walls^4,7–9^.

During the last years, several studies highlighted the potential benefits of postnatal treatment with melatonin to cope PAHN, showing that it has effective antioxidant, vasodilator, antiremodeling and antihipertensive effects at the pulmonary level^3,5,6,9,10^. Although melatonin decreased large arteries thicknesses, such as the main pulmonary and aorta arteries, no biomechanical changes has been observed^2^. However, few is known still about the biomechanical changes that take place on the major arteries of the circulatory system due to melatonin administration^2^.

In this context, there is great interest in evaluating the mechanical response of the arteries of animals affected by PAHN^3^, since this biomechanical knowledge becomes an essential information for the development of proper diagnostics and treatment in cardiovascular and cardiopulmonary impairments^11,12^.

An important aspect that must be developed in biomechanical studies is the mechanical characterization of biological tissues, where it is necessary to choose a constitutive model able to properly define specific characteristics between materials^13,14^. A passive behavior analysis usually considers an elastic rate-independent material response, under the assumption of quasi-incompressibility^15,16^. While several authors have characterized the arterial response using isotropic models which do not consider the structure of the arterial wall^17–20^, others have incorporated some microstructural characteristics (i.e., stiff structure provided by the collagen fibers) present in the blood vessels motivated by the intrinsic anisotropic response^14,16,21,22^. The formulations of the constitutive models were developed within the framework of the mechanics of the nonlinear continuum, and then implemented in the context of the finite element method (FEM)^21,23,24^.

In order to characterize a hyperelastic material it is necessary to fit the experimental data from different mechanical tests to a hyperelastic constitutive model. The association between the experimental data and the mathematical model is done through an objective function that needs optimization. It is noteworthy to mention that several methods have been employed to characterize the constitutive parameters of a hyperelastic material^25–27^. Moreover, different authors have used the Least-Squares regression method, which is based on an iterative algorithm that requires the definition of an initial condition and the differentiation of an objective function. Another method widely used is the Levenberg–Marquardt Method (LMM); however, it only performs a local search for the optimum and its convergence is highly dependent on the initial condition^28^. To find the parameters of an anisotropic hyperelastic model, another option is the use a meta-heuristic optimization technique that belongs to the set of evolutionary strategies (ES)^29^. The main reason for this choice is that ES useful in solving inverse problems, such as retrieving the constitutive parameters of a model, in different scientific domains^30–32^. Furthermore, in contrast to Gradient Descent methods, ES are not local optimisation techniques and they are less sensitive to initialisation^33^. Also, ES do not require knowledge of any initial condition or the gradients of a function^34^. In addition, there is not need to compute complicated derivatives nor to define restrictions when aplying ES^35^. The only requirement is to define the search space and an objective or fitness functional involving the parameters to be retrieved. In summary, ES seem well suited for the characterization of non-linear materials with multiple constraints.

The objective of the present work is to characterize the passive mechanical properties of the aorta artery of lambs gestated and born under chronic hypoxia at high altitude (3600 masl) suffering from PAHN. To that end, we have structured this contribution as follows. We firstly describe the materials (lamb thoracic aorta) for two experimental groups, control and melatonin-treated. To assess biomechanical characteristics, we applied tensile and pressurization tests in aortas. We also present the Gasser–Holzapfel–Ogden (GHO) hyperelastic anisotropic constitutive model used for the mechanical characterization whose set of parameters was calibrated simultaneously from the tensile and pressurization tests measurements using an evolutionary strategy which is an algorithm oriented to global optimization. Finally, we describe the geometry, the boundary conditions, and the computational simulation of the pressurization test that includes the physiological measurements of axial stretch test.

## Material and methods

### Material

The tissue studied in this work corresponded to thoracic aortas (TA) of 9 newborns lambs gestated at high altitude (Putre, Chile, 3600 masl). In this altitude level, the animals developed PAHN due to their gestation and birth under chronic hypobaric hypoxia^2,3,5,6^.

The lambs were randomly assigned to one of the two experimental groups, one control and other melatonin-treated. In the control group (CN, $n=5$), lambs were administered with the vehicle orally (1.4% ethanol 0.5 ml kg$^{-1}$ per day). In the melatonin group (MN, $n=4$), lambs received oral melatonin (melatonin 1 mg kg$^{-1}$ in ethanol 1.4%, 0.5 ml kg$^{-1}$ per day, during 21 days, at dusk (20:00 h) to follow the circadian rhythm of melatonin). The dose of melatonin administered is sufficient to increase the plasma level approximately nine times without affecting the diurnal plasma concentration of this hormone^6,10^.

All animal care, procedures and experimentation were approved by the Ethics Committee of the Faculty of Medicine, University of Chile (protocol CBA#0761 FMUCH), and were conducted in accordance with the U.K. Animals (Scientific Procedures) Act, 1986 and the ARRIVE guidelines (https://arriveguidelines.org).

### Biomechanical tests

#### Tensile test

The uniaxial tensile test applied on soft tissues is commonly used for characterizing the physical properties of the artery wall^16,20,36^. It constitutes a simple and easy to implement in vitro procedure to obtain the mechanical properties of tissues. However, it is insufficient to achieve a robust mechanical characterization, since it does not allow to replicate the complex loading conditions present in a physiological state^37,38^. Its main result is the stress-stretch relation under uniform deformation and it provides a set of parameters that have been used to compare the mechanical responses on soft tisues^2,16,39,40^.

It is important to highlight that the experimental results reported by Rivera et al.^2^ were used in this work in the characterization procedures of the thoracic aorta artery. In the aforementioned work, the samples were analyzed immersed in calcium-free saline (Krebs), at 39$^\circ$C. The stress–stretch relationship was obtained by calculating the Cauchy axial stress as $\sigma =\frac{F}{A}$ where *F* is the axial load and *A* is the instantaneous cross sectional area (where the quasi-incompressibility condition is considered) and the axial stretching as $\lambda =\frac{L}{L_0}$ where *L* and $L_0$ are the instantaneous and initial measurements of the sample, respectively^2,16^.

Some biomechanical parameters of interest that allow comparing the tensile mechanical behavior in the physiological function range are the distensibility and the incremental module^11^. The distensibility is given by:

1 $$\begin{aligned} DC=\dfrac{D_s^2-D_d^2}{D_s^2 (P_s-P_d)} \end{aligned}$$

where $D_d$ and $D_s$ are the diameters in diastole and sistole, respectively, while $P_d$ and $P_s$ are the pressures in diastole and sistole, which for the lamb model correspond to 60 and 120 mmHg, respectively^2,41^.

The incremental module represents the secant of the stress-stretch curve in the physiological pressure values (systolic and diastolic) and is given by:

2 $$\begin{aligned} E_{inc}=\dfrac{3}{DC} \dfrac{4t+D_s}{4t} \end{aligned}$$

where *t* is average initial unloaded arterial wall thickness^11^.

Finally, as detailed below, the experimental uniaxial tensile test results are considered in this work to obtain the material parameters that determine the mechanical behavior of the studied arteries by means of an anisotropic constitutive model which is later used in turn to estimate the circumferential stresses inside the arterial wall via computational simulation.

#### Pressurization test

This test is intended to replicate the *in vivo* load of a blood vessel. In this test, a portion of a blood vessel is subjected to an axial stretch in the tensile machine followed by the application of internal pressure by means of a fluid (calcium-free saline) that is run through to the inside of the vessel to enact radial loading^36,42,43^. For this purpose, the ends of the cylindrical samples of the thoracic aorta are fixed to metallic nozzles. The adopted set-up of the pressurization test is similar to that already described in^36^. That is, the samples permanently submerged in physiological serum (Krebs calcium free), at temperature of 39±1$^\circ$C. In order to achieve uniform temperature in the sample, a time interval of 10 min with the submerged sample was considered before starting the test.

**Figure 1:**
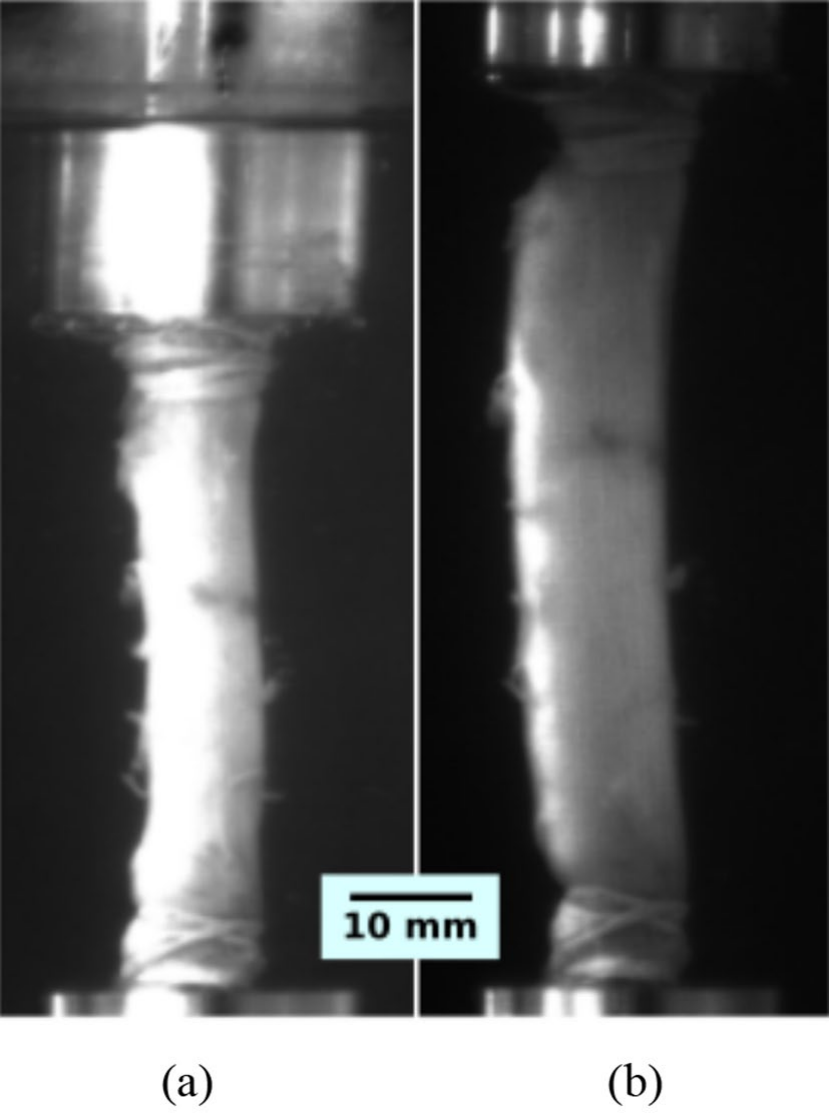
Experimental assembly of the pressurization test. (**a**) Thoracic aorta without stretching and free of loads, immersed in Krebs; (**b**) Pressurization of a previously stretched thoracic aortic artery.

The test was carried out in two steps. The first step was an axial stretch of the artery to its physiological length, $\lambda _z$, which is kept constant during the test. The axial stretch is calculated as $\lambda _{z}= \frac{L}{L_0}$ where *L* was the initial or physiological lengths and $L_0$ is the measurements after the artery was extracted. In this work, we used the value obtained from the first three sections of the thoracic aorta as reported by Rivera et al.^2^ which, for the control (CN) and melatonin (MN) group, has an average of $\lambda _{z}=1.20$. The second step was the pressurization. The internal pressure and external diameter of the vessel were obtained during the whole test using video recordings, which were then processed to plot the internal pressure vs. diametral stretch curve. The diametral stretch was defined as $\frac{D}{D_0}$ , where *D* and $D_0$ denote the current and initial diameters of the vessel, respectively. In this work, 10 successive loading cycles were run up to a pressure of 170 mmHg to precondition the samples. The last cycle was used to perform the inflation analysis. Figure 1 shows the experimental assembly used in this work.

### Constitutive modelling

According to the measurements of the tensile tests, an elastic rate-independent material response was considered for the analyzed arteries. Furthermore, a quasi-incompressible arterial behavior was assumed due to the large amount of water present in them, evidenced by a small volume change during deformation and analytical simplification of the arterial wall’s elasticity^15,44,45^. On the other hand, it is important to note that there are studies which suggest considering the compressibility of the material, that is between 2 and 6 %^46^. However, this issue does not yet hold a definitive answer^46,47^. With the purpose of characterizing the mechanical response of the biological tissue, use is made of the constitutive hyperelastic model of Gasser–Holzapfel–Ogden (GHO)^24^, whose deformation energy function *W* describes the behavior of the isothermal material under any load condition and can be defined in terms of the right Cauchy deformation tensor $\mathbf {C} = \mathbf {F}^{T} \mathbf {F}$, where $\mathbf {F}$ is the deformation gradient tensor and *T* is the transpose symbol. Using classical arguments of continuum mechanics, the Cauchy stress tensor is defined as $\sigma = 2 J^{-1} \mathbf {F} \frac{\partial W}{\partial \mathbf {C}} \mathbf {F}^T$, where *J* is the determinant of $\mathbf {F}$.

**Figure 2:**
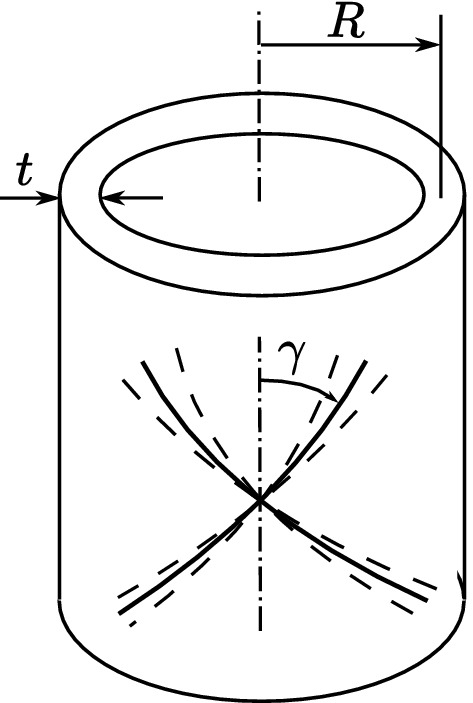
Representation of arterial wall with two embedded families of fibres. The mean orientations of the collagen fibres are characterized by the angle $\gamma$.

The GHO model describes an anisotropic response of the arteries, since it includes the action of the collagen fibers that give the material a great resistance to traction. The functional form of the model, when considering a volumetric part and an isochoric part, is given by:

3 $$\begin{aligned} W= & {} \dfrac{p}{2}(\ln J)^2 + \frac{\mu }{2} (\bar{I}_1-3) + \frac{k_1}{2 k_2} \sum _{i=1,2} \left[ \exp \left\{ k_2 [\kappa \bar{I}_1 +(1-3 \kappa )\bar{I}_{4i}-1]^2 \right\} - 1 \right] \quad \end{aligned}$$

where *p* is the hydrostatic pressure, which must be evaluated based on boundary conditions, and acts as a penalty term to ensure incompressibility of the tissue^23,48^, $\bar{I}_1=\bar{\mathbf{C }}:\mathbf{I} =tr(\bar{\mathbf{C }})$ is the first invariant of $\bar{\mathbf{C }} = J^{-2/3} \mathbf{C}$ and the invariants $\bar{I}_{4i}$, with $\ i=1,2$, are defined as $\bar{I}_{41} = \bar{\mathbf{C }}:(a_1 \otimes a_1)$ and $\bar{I}_{42} = \bar{\mathbf{C }}:(a_2 \otimes a_2)$ such that $a_1$ and $a_2$ are two unit vectors defined in the reference configuration and arranged at a $\pm \gamma$ angle with respect to the axis of the vessel, which takes into account the orientations of two families of symmetric fibers. Furthermore, $\mu$, $k_1$, $k_2$ and $\kappa$ are material parameters (all of them with positive values). The parameter $\kappa$ is associated with the dispersion of the fibers, taking values between 0 and 1/3^24^. In Eq. (3), the first term is the volumetric component; the second term corresponds to a classical isotropic Neo-Hookean model, while the third and fourth terms have the objective of predicting the anisotropic behavior and the rigidization of the artery wall due to the action of the collagen fibers distributed symmetrically around the arterial wall, as illustrated in Fig. 2. This model is aimed at characterizing consistently the anisotropy of the material present in the arterial tissues when they are subjected to moderate to high deformation levels. For simplicity, it must be kept in mind that specific responses of each arterial layer are not described by this model.

### Numerical simulations

#### Pressurization test

The pressurization test is an excellent complement to uniaxial tensile tests when characterizing and establishing parameters of a constitutive model. Additionally, the numerical simulation of this test allows evaluating the variation of the circumferential stresses at different levels of internal pressure, allowing to analyze extreme conditions and states of arterial hypertension.

**Figure 3:**
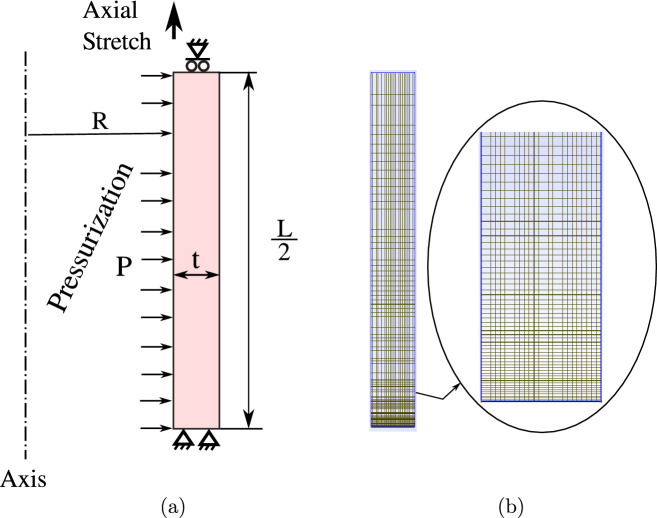
(**a**) Representation 2D of the boundary conditions for the computational simulation of the pressurization test. (**b**) View of the 2D finite element mesh.

In this work, the finite element analysis of this test corresponds to a 2D axisymmetric simulation, since the symmetries that come from assuming a perfectly cylindrical initial artery are considered. The simulation is made up of two fundamental steps. The first step is an axial stretch of the artery to its physiological length. The physiological elongation corresponds to a value of $\lambda _z = 1.20$, which is kept constant during the simulation of the pressurization stage. The second step is the pressurization process (inflation), where an internal pressure is applied to the arterial wall that covers the physiological range and extends to hypertension values, up to 170 mmHg. Figure 3a summarizes this loading procedure.

The computational reconstruction corresponds to a rectangular surface (or longitudinal section) obtained by making a longitudinal cut to a cylindrical tube. The dimensions that define the geometry, i.e., the internal radius and thickness, were gathered from the average measurements of the thoracic aortic arteries collected from ring opening tests. Meanwhile, the length was defined as the average of the samples retrieved from the pressurization test. The dimensions corresponding to the internal radius, thickness and length of both groups were for CN: $R_0=3.57 \ mm$, $t=2.06 \ mm$, $L=14 \ mm$ and for MN: $R_0=3.26 \ mm$, $t=2.14 \ mm$, $L=14 \ mm$.

The mesh shown in Fig. 3b has 2626 nodes that correspond to $25 \times 100 = 2500$ elements (quadrilaterals). This mesh contains a refinement towards the base, where the artery is anchored, in order to properly capture the edge effects expected in this zone.

### Material parameters calibration procedure

#### Objective function

In order to model the arteries according to the GHO constitutive model^24^, it is necessary to calibrate its parameters. For this purpose, different mechanical tests are carried out on this material. Thus, it is necessary to propose a calibration procedure that maximizes, through the definition of an objective function, the similarity between the behavior of the hyperelastic model and the experimental data and also guarantees a stable response of such model.

By considering multiple mechanical tests in the characterization process, it is possible to capture the mechanical behavior of the arteries under different modes of deformation. Therefore, experimental measurements collected from the tensile (along both the longitudinal and circumferential directions) and pressurization tests are used in this work in order to obtain the material parameters of the GHO model.

Firstly, it should be noted that it is not possible to obtain a closed analytical expression of the Cauchy stress $\sigma _1$ for the GHO constitutive model when the material is subjected to uniaxial tensile stress. According to the procedure defined by Ogden^49^, the mechanical response in this particular case can be determined by the following mathematical expressions:

4 $$\begin{aligned}\sigma _{1}(\mathbf {x},\lambda _1,\lambda _2)=2(\lambda _1^{2}-\lambda _1^{-2}\lambda _2^{-2})W_1+2\lambda _1^2 \cos (\gamma -\beta )^2W_4+2\lambda _1^2 \cos (\gamma +\beta )^{2}W_6 \end{aligned}$$

5 $$\begin{aligned}\sigma _{2}(\mathbf {x},\lambda _1,\lambda _2)=2(\lambda _2^{2}-\lambda _1^{-2}\lambda _2^{-2})W_1+2\lambda _2^2 \sin (\gamma -\beta )^2W_4+2\lambda _2^2 \sin (\gamma +\beta )^{2}W_6=0 \end{aligned}$$

where $W_i = \frac{\partial W}{\partial I_i} \ (i=1,4,6)$ and $\beta$ denotes the sample orientation (i.e., $\beta =0^{\circ }$ and $90^\circ$ for longitudinal and circumferential samples, respectively). It is possible to observe that $\sigma _{1}$ depends on $\lambda _1$ and $\lambda _2$ so, it is necessary to use Eq. (5), for a given set of parameters $\mathbf {x}=(\mu ,\kappa ,k_1,k_2,\gamma )$, in order to find the value of $\lambda _2$. This last operation is done through the Newton–Raphson method due to the nonlinear nature of the equation.

**Figure 4:**
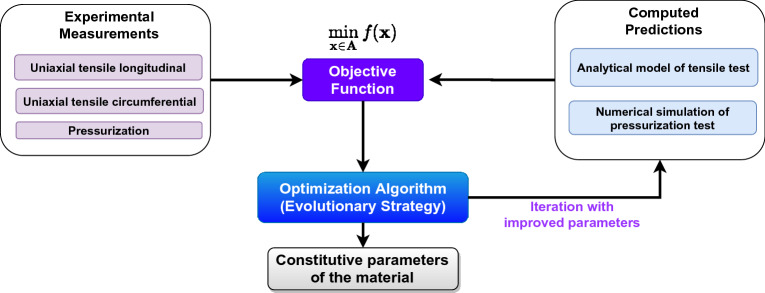
Algorithm of the inverse problem-solving method. The evolutionary loop shows the main steps of the optimization problem.

Secondly, the mechanical behavior of the pressurization test is modeled using the numerical approach presented in “Pressurization test” since analytical expressions describing the material response in this test are only limited to certain conditions that are not met in this case^50^. To calculate the standardize quadratic errors of each of the mechanical tests, the following function can be defined:

6 $$\begin{aligned} J(\varvec{y},\hat{\varvec{y}})=\frac{1}{n}\sum _{i=1}^{n} \frac{(y_i-\hat{y_i})^2}{|max(\varvec{y})-min(\varvec{y})|} \end{aligned}$$

where *n* is the number of experimental points, $\varvec{y}$ are the experimental values and $\hat{\varvec{y}}$ are the values predicted by the model. Forty sampling points ($n=40$) were used to capture the behavior of each mechanical test since this is sufficient to capture the continuous and monotonically increasing behavior of them. With this metric, it is possible to define the following objective function:

7 $$\begin{aligned} \min _{\mathbf {x}\in \mathbf {A}}f(\mathbf {x})=J(\varvec{\sigma }^{circ},\hat{\varvec{\sigma }}^{circ})+J(\varvec{\sigma }^{long},\hat{\varvec{\sigma }}^{long})+2J(\varvec{\lambda }_{\theta }^{presu},\hat{\varvec{\lambda }}_{\theta }^{presu}) \end{aligned}$$

where $\varvec{\sigma }^{circ}$ and $\varvec{\sigma }^{long}$ are the uniaxial stress for the circumferencial and longitudinal tensile curves, respectively and $\varvec{\lambda }_{\theta }^{presu}$ are the diametral stretches of the presurization test. The proposed objective function is mono objective; therefore, it is essential to weigh the error of each of the curves correctly. For this purpose, function 6 was introduced to standardize the curves by the number of data points and the experimental range. The pressurization test weighs the same as the two uniaxial tests because it is the leading arterial deformation state which is the objective of this work. In addition, this response comes from a numerical simulation that considers the inhomogeneous deformation gradients and enriches the characterization process. It is possible to choose other weights, but it is not within the scope of this study. This objective function is restricted to a stability domain given by the set $\mathbf {A}$ that is defined as:

8 $$\begin{aligned} \mathbf {A}=\left\{ \varvec{x}\in \mathbb {R}^5\mid \kappa \in ]0,1/3] \, \wedge \, \gamma \in [0,\frac{\pi }{2}]\, \wedge \, \frac{d\Psi }{d\lambda _1}- \frac{7\Psi }{5\lambda _1}<0 \,\forall \, \lambda _1 \in \left\{ \lambda _{i}^{circ},\lambda _{i}^{long} \right\} \right\} \end{aligned}$$

where $\Psi =W_1^{-1}[W_1+\sin (\gamma +\beta )^2W_4+ \sin (\gamma -\beta )^2W_6]$. For more details about this stability criterion, please refer to Canales^51^. Lastly, the evolutionary strategies (ES)^52^ are used to solve the nonlinear constrained optimization problem defined in Eq. (7) which serves as a link between the inversion method and the physics of the material that is being studied. The whole material parameters calibration procedure is summarized and illustrated in Fig. 4.

#### Evolutionary strategies

The optimization problem presented in Eq. (7) has a non-linear nature that is subjected to multiple constraints. ES is an algorithm oriented to global optimization that has proven to be a suitable and versatile tool to solve these kind of problems^53,54^ and therefore, were used in this work. A brief description of them is presented below, for more details, we refer the interested reader to the excellent work of Macías et al.^55^ or the book of Beyer^56^.

The initial step before to the beginning of the optimization process is the random initialization of an assembly of vectors $\mathbf {x}$ that will constitute the initial population $P_{\mu }^{(g)}|_{g=0}$, where $\mu$ is the number of elements within the initial population and *g* is the generation of the population which is associated with the iteration of the algorithm. A canonical evolutionary optimization algorithm is based on the application, over a defined number of iterations, of two genetic operators with well defined roles. The first is the recombination, which exploits the search space through the exchange of information between $\rho$ different elements of the population. The second operator is the mutation, which is used to explore the search space through the introduction of random variations in the population. The application of these genetic operators over the initial population leads to the generation of a secondary population $P_{\lambda }^{(g)}$ of $\lambda$ elements. It is at this stage of the evolutionary loop that the link between the physics of the problem studied and the optimization algorithm is established. In the present work, this is done through the minimization of the functional defined by Eq. (7), which can also be interpreted as a measure of the similarity between the experimental data and the mechanical response of the hyperelastic constitutive model. Each element of the secondary population will be evaluated, and only those elements of $P_{\lambda }^{(g)}$ that minimize the objective function will be retained, through some selection scheme, as part of the population $P_{\mu }^{(g+1)}$ for the next iteration of the evolutionary loop. The procedure is repeated until a defined stopping criteria has been fulfilled. The respective sizes of the initial and the secondary populations, $P_{\mu }^{(g)}$ and $P_{\lambda }^{(g)}$, remain constant throughout the entire search process. In this work, the elitist selection (ES -$(\mu /\rho +\lambda ))$ is used, which selects the junction of the initial and mutated populations. Consequently, a promising element belonging to the first initial population can survive throughout the entire optimization process. Although this attribute of the ES guarantees a monotonic decrement or increment of the fitness function, it can also make it prone to a premature convergence into a local optimum.

It is important to emphasize that this type of algorithm is oriented to the global optimization of a problem and is not particularly sensitive to initialization^57^. In this work, we use a total population of 280 random individuals and 100 generations to avoid stagnation in a local optimum.

### Statistic analysis

All the data were expressed as the *means* ± *standard error of the mean* (SEM), which is the ratio between the standard deviation and the square root of the number of specimens. The results were compared statistically by means of the non-parametric Mann-Whitney test for independent random samples. Significant differences were accepted when $p \le 0.05$ (Prism 5.0; GraphPad).

## Results and discussion

### Material characterization via tensile and pressurization tests

**Figure 5:**
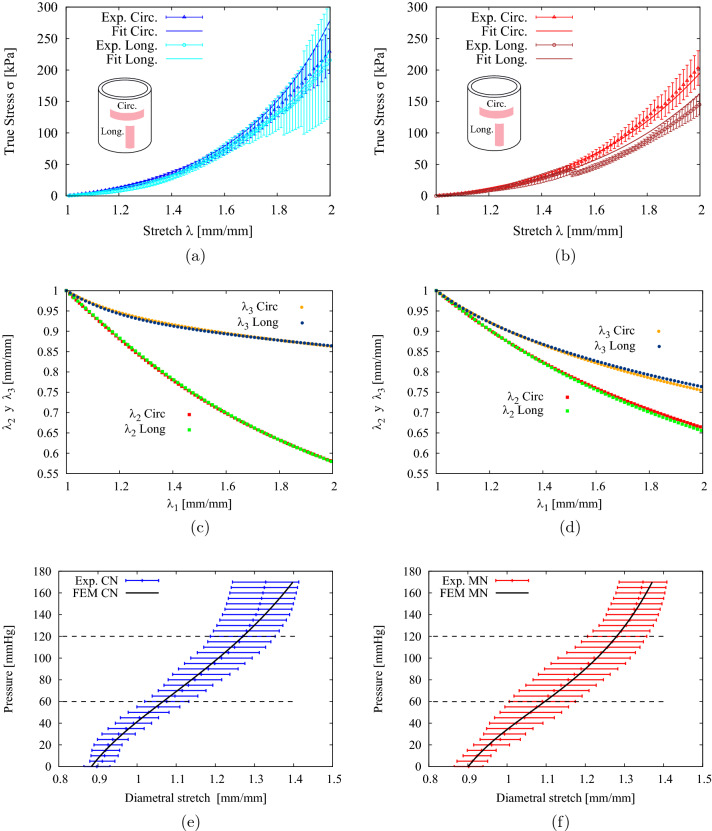
(**a**,**b**) Thoracic aorta characterization via Cauchy stress-stretch curves for control (CN) and melatonin (MN) group lambs. The fitting were compute via GHO’s model. (**c**,**d**) Realistic numerical results of the GHO model tensile stretchs for the aorta artery. (**e**,**f**) FEM numerical simulation of the pressurization test.

Figure 5a,b present the graphs of the average stress-stretch curves of the thoracic aorta for the control (CN) and melatonin (MN) groups, respectively. Each figure contains the experimental data for the circumferential and longitudinal directions (the vertical bars indicate the standard error of the mean, SEM). It is seen that all the curves present a typical hyperelastic behavior, that is, a first linear stage followed by a transition stage, and finally a linear (rigidization stage).

Furthermore, as already mentioned, the experimental results of the tensile tests were used to determine material parameters of the anisotropic constitutive model of GHO described in “Constitutive modelling”. For simplicity, the material parameters were derived from each pair of average curves (longitudinal and circumferential) corresponding to each group, applying an evolution strategies (see “Evolutionary strategies”). The analytical expressions of the stress field which assume flat stress conditions used to derive the material parameters, given explicitly in the paper by Garcia-Herrera et al.^16^. The values of the material parameters obtained are presented in Table 1. The curve corresponding to the fit is presented by means of the GHO constitutive model, using the values of the parameters and they provide physically motivated responses^58,59^ as can be seen in Fig. 5c,d. If we compare the parameters of the arterial groups (CN and MN), it is seen that the resultant values are similar and fit the experimental curves with sufficient precision, as shown by the values of the fitting quality for each curve (see Fig. 5a,b). The correlation indicator used was Efron’s Pseudo R-Squared ($r^2$), which is shown in Table 1.

**Table 1:** Material parameters of the GHO model for aorta artery and correlation indicators for tensile curves ($r_{circ}^2$ and $r_{long}^2$) and pressurization curves ($r_{press}^2$).

Artery	$\mu$ [*kPa*]	$k_1$ [*kPa*]	$k_2$	$\kappa$	$\gamma [^o]$	*p* [*kPa*]	$r_{circ}^2$	$r_{long}^2$	$r_{pressu}^2$
TA CN	16.25	24.18	0.233	0.123	46.38	$16 \cdot 10^3$	0.956	0.992	0.976
TA MN	16.58	27.97	0.757	0.253	49.23	$16 \cdot 10^3$	0.994	0.959	0.996

### Assessment of the pressurization test

Figure 5e,f shows pressure vs. diametral stretch curves corresponding to groups CN and MN for a value of axial stretch $\lambda _z=1.20$. The experimental response of both groups is similar and there is no evidence of significant differences. The internal pressure curve vs. diametral stretch curves obtained with the GHO model via computational simulation FEM are also presented in aforementioned figures. The horizontal lines denote the physiological range of diastole and systole of the cardiac cycle, which was estimated between 60 mmHg and 120 mmHg. The distensibility and incremental module measurements presented in Table 2 show similar biomechanical response in the physiological pressure range between the MN group and the CN group and no present significant changes. In the same table, by way of comparison, the values obtained by Rivera et al.^2^ from the tensile tests are presented, it can be seen that these measurements show a similar biomechanical response.

**Table 2:** Distensibility, *DC*, and incremental modulus of elasticity, $E_{inc}$, obtained in the tensile by Rivera^2^ and pressurization tests obtained in this work, with axial stretch $\lambda _z=1.20$. Values were expressed as Mean ± SEM. Significant differences ($P\le 0.05$): * vs. CN.

Thoracic Artery	Pressurization test results	
	$DC \ [mmHg^{-1}]\times 10^{-3}$	$E_{inc} \ [MPa]$
CN	5.53±0.47	0.241±0.029
MN	5.51±0.41	0.224±0.021

Thoracic Artery	Tensile test results	
	$DC \ [mmHg^{-1}]\times 10^{-3}$	$E_{inc} \ [MPa]$
CN (Rivera 2020)	4.63±0.36	0.249±0.022
MN (Rivera 2020)	4.65±0.43	0.220±0.024

Also in the Table 3 is presented the circumferential stress for three different loading conditions (pressures 60, 120 and 170 mmHg). The comparison between the CN and MN groups was made by means of circumferential stresses of the inner and outer faces ($\sigma _{\theta \ int}$ and $\sigma _{\theta \ out}$ respectively), its difference ($|\Delta \sigma |=|\sigma _{\theta \ int}-\sigma _{\theta \ out}|$) and its average ($\sigma _{mean}= (\sigma _{int}+\sigma _{out})/2$)^16,40^. The comparison between the MN and CN groups in general do not present significant changes.

**Table 3:** Circumferential stress from the simulation of the presurization on the thoracic aorta for pressures $P_i=60, \ 120$ and 170 mmHg. The simulation added fisiological axial stretch $\lambda _z=1.20$.

Thoracic Artery	Group	$\sigma _{\theta \ int}$ [*kPa*]	$\sigma _{\theta \ out}$ [*kPa*]	$|\Delta \sigma _{\theta }|$ [*kPa*]	$\sigma _{\theta \ mean}$ [*kPa*]
$P_i=60$ mmHg	CN	34.303	17.234	17.069	25.769
	MN	41.173	15.602	25.571	28.388
$P_i=120$ mmHg	CN	140.51	46.361	94.149	93.436
	MN	176.29	38.304	137.99	107.30
$P_i=170$ mmHg	CN	297.81	74.281	223.53	186.05
	MN	366.34	55.075	311.27	210.71

In this work, we evaluate the effects of melatonin, through circumferential stresses, distensibility and incremental modulus. Previous studies have shown structural and functional effects of melatonin in neonatal sheep with PAHN^2,6,9,10^. Therefore, we hypothesized that melatonin modifies the structure and biomechanical characteristics of the thoracic aorta. Our study shows that an oral administration of 1 mg kg$^{-1}$ per day of melatonin does not have significant effects on the mechanics of the thoracic aortic wall (Tables 2 and 3).

## Conclusion

The experimental work, the modeling and the numerical simulations based on the uniaxial tensil and pressurization tests on aorta artery samples have been presented.

The main objective of this study was the biomechanical characterization of the aorta artery in lambs gestated and born at high altitude (3600 masl), affected by pulmonary arterial hypertension in newborns (PAHN).

Experimental data from tensile tests and pressurization tests have been used to determine the material parameters of the Gasser–Holzapfel–Ogden hyperelastic anisotropic constitutive model, where simultaneously the constitutive modelling the tensile (on two direction) and the pressurization test have been experimentally validated, obtaining high goodness of fit in curves. The evolutionary strategy procedure used ensures the stability of the constitutive model and as a consequence, physically motivated responses are obtained.

The application of an evolutionary strategy to samples of an animal lamb model is an original contribution of this research.

In addition, we have shown that a postnatal melatonin treatment does not modify the biomechanical properties of the thoracic aorta in lambs gestated and born under chronic hypoxia.

Finally, it is important to mention that the proposed biomechanical characterization is versatile and allows the inclusion of viscous and damage effects through new terms in the objective function. The latter is a limitation of the present study, together with the inclusion of some microstructural characteristics.
